# Extended perfusion protocol for MS lesion quantification

**DOI:** 10.1515/med-2020-0100

**Published:** 2020-06-08

**Authors:** Eleftherios Kontopodis, Kostas Marias, Georgios C. Manikis, Katerina Nikiforaki, Maria Venianaki, Thomas G. Maris, Vasileios Mastorodemos, Georgios Z. Papadakis, Efrosini Papadaki

**Affiliations:** Foundation for Research and Technology – Hellas, Institute of Computer Science, Computational Bio-Medicine Laboratory, N. Plastira 100, Vassilika Vouton, GR-700 13 Heraklion, Crete, Greece; Department of Radiology, Medical School, University of Crete, P. O. Box 2208, Heraklion, Crete, Greece; Technological Educational Institute of Crete, Department of Informatics Engineering, Heraklion , Crete, Estavromenos, TK 71410, Greece; Science and Technology Park of Crete, Gnosis Data Analysis, N. Plastira 100, Vassilika Vouton, GR-700 13, Heraklion, Greece; Department of Neurology, Medical School, University of Crete, P. O. Box 2208, Heraklion, Crete, Greece

**Keywords:** DCE-MRI, perfusion protocol, MS, active demyelinating lesions

## Abstract

This study aims to examine a time-extended dynamic contrast-enhanced magnetic resonance imaging (DCE-MRI) protocol and report a comparative study with three different pharmacokinetic (PK) models, for accurate determination of subtle blood–brain barrier (BBB) disruption in patients with multiple sclerosis (MS). This time-extended DCE-MRI perfusion protocol, called Snaps, was applied on 24 active demyelinating lesions of 12 MS patients. Statistical analysis was performed for both protocols through three different PK models. The Snaps protocol achieved triple the window time of perfusion observation by extending the magnetic resonance acquisition time by less than 2 min on average for all patients. In addition, the statistical analysis in terms of adj-*R* ^2^ goodness of fit demonstrated that the Snaps protocol outperformed the conventional DCE-MRI protocol by detecting 49% more pixels on average. The exclusive pixels identified from the Snaps protocol lie in the low *k* ^trans^ range, potentially reflecting areas with subtle BBB disruption. Finally, the extended Tofts model was found to have the highest fitting accuracy for both analyzed protocols. The previously proposed time-extended DCE protocol, called Snaps, provides additional temporal perfusion information at the expense of a minimal extension of the conventional DCE acquisition time.

## 1 Introduction

Multiple sclerosis (MS) is a chronic inflammatory demyelinating disease of the central nervous system (CNS) usually affecting young adults. Although the etiology of MS is largely unknown, it is considered primarily an autoimmune disease in which activated myelin-specific T-cells migrate from the periphery to the CNS, by crossing the blood–brain barrier (BBB), and induce the formation of new inflammatory demyelinating lesions [1,2]. Recent studies have emphasized the crucial role the BBB dysfunction plays in the inflammatory events that take place in MS [3,4]. Histopathological and magnetic resonance imaging (MRI) studies reported BBB abnormalities not only in acute active inflammatory MS lesions but also in inactive, non-enhancing lesions and the normal appearing white matter (NAWM) as well [5,6,7]. According to research studies concerning the development of drug therapies in MS, the leukocyte passage across the BBB is very important for disease pathophysiology [8,9] and resolution of inflammation along with the protection of BBB function is the therapeutic target for many proposed MS treatments [1,2]. So, developing quantitative MRI techniques that detect and quantify BBB permeability is of paramount importance in understanding the pathophysiology, determination of disease activity, and estimation of treatment efficacy in MS.

Dynamic contrast-enhanced magnetic resonance imaging (DCE-MRI) is a quantitative MRI technique able to detect and quantify the disruption of the BBB. It comprises the dynamic acquisition of multiple T1-w images before, during, and after the administration of a paramagnetic contrast agent (CA) [10,11]. Many compartmental models have been proposed to quantify the CA pharmacokinetics (PK), from very simple implementations that assume a single compartment, to models incorporating more complex assumptions [11].

There are several studies in the literature that investigate the integrity of the BBB in patients with MS using the DCE-MRI technique [10,12,13]. Considering the low enhancement appearing in the MS lesions and the peripheral NAWM, some studies have been focusing on examining the acquisition and protocol parameters as well as the model selection in order to have a more robust quantification of the BBB disruptions in the aforementioned areas. It has been reported that for accurate quantification of subtle BBB permeabilities, the Patlak model is the most appropriate [5,14], while for higher permeabilities a more complex model, such as the extended Tofts model (ETM), should be used [5]. Moreover, it has been reported that long overall perfusion acquisition time and long baseline acquisition will result in a more accurate measurement of subtle BBB leakages [14]. Finally, in another study of Jelescu et al. [15], a dual temporal resolution protocol was proposed in order to improve measurement accuracy and precision. This protocol consisted of an initial part at a high temporal and low spatial resolution, lasting for 1 min, in order to better capture the first-pass bolus. The second part consisted of low temporal resolution and high spatial resolution, essential to properly detect and segment the active MS.

To this end, and considering reports from previous studies on low BBB leakage on visibly non-enhancing lesions, NAWM of MS and healthy brain WM [16,17], more recent studies have tried either to quantify [18,19,20] or to just detect [6] these subtle BBB disruptions through the use of DCE-MRI.

Finally, it is of great interest to report three previous studies that tried to quantify BBB abnormalities on MS, by also examining the late dynamics of the signal enhancement. Gaitán et al. studied 80 patients with relapsing–remitting MS (RRMS) by examining the morphological features of the enhancement patterns up to 60 min after CA administration [21]; Soon et al. examined 19 patients with MS by investigating the T1 longitudinal relaxation times up to 60 min after CA administration [22], while Shinohara et al. analyzed 10 patients with MS by exploring the lesion enhancement curves using a functional principal component analysis up to 155 min after CA administration [23].

One of the limitations of the aforementioned studies is the fact that some of them utilized qualitative or semi-quantitative PK analysis [6,21,22,23], while others did not pay a lot of attention to the duration of the perfusion protocol [18,19,20]. The aim of this study was to further examine a previously presented DCE-MRI framework, which included a newly introduced protocol, as well as a method for the selection of a suitable PK model for the accurate quantification and detection of even subtle disruption of BBB in MS lesions, by minimally extending the conventional DCE acquisition time. This study follows a preliminary study that included a small dataset of four patients with RRMS [24]. In the current study, a larger cohort of patients were examined, and the data analysis was more specific in terms of considering each enhancing lesion separately and also examining the peripheral lesion tissue.

## 2 Materials and methods

### 2.1 Patient information and imaging protocol

In the current study, 56 consecutive patients with RRMS (42 females) with mean age of 35.9 ± 10 years were initially scanned. Twelve patients (8 females) with active disease, as proved by the existence of contrast-enhanced focal demyelinating lesions, were further included in the analysis. The mean age at disease onset was 30.3 years, mean age at diagnosis was 30.8 years, and mean disease duration was 3.7 years (Table 1). Research methods in the current study complied with all the relevant national regulations, institutional policies and in accordance with the tenets of the Declaration of Helsinki and were approved by the corresponding institutional review boards. The procedure was thoroughly explained to all patients who signed the informed consent. All examinations were performed on a 1.5 T MR scanner (Hybrid Vision/Sonata, Siemens/Erlangen, Germany). Given that quality assurance (QA) protocols are embedded in a routine QA program for the specific head coil, it can be stated that signal-to-noise ratio (SNR) is greater than 100, when using phantom measurements (ACR100), with tolerance levels at <5% on a yearly basis. Under this rationale, this can imply that measurements stemming from the proposed protocol do not suffer from signal fluctuation or temporal signal drift.

**Table 1:** Patient and protocol information

Pt number	Sex	Age	# active lesions	# pixels	# Snaps acquired	DCE acquisition time (min:s)/window time of perfusion observation (min:s)	
						NoSnaps	Snaps
1	f	33	2	173	8	6:00/6:00	7:30/26:00
				356			
2	f	24	10	164	6	6:00/6:00	7:08/23:00
				1,939			
				201			
				100			
				247			
				258			
				1,239			
				365			
				146			
				277			
3	f	16	2	252	9	6:00/6:00	7:43/27:00
				2,651			
4	f	32	1	375	6	6:00/6:00	7:08/24:00
5	f	25	1	1,561	7	6:00/6:00	7:19/25:00
6	m	55	2	414	6	6:00/6:00	7:08/23:00
				1,072			
7	f	34	1	2,991	8	6:00/6:00	7:30/26:00
8	f	29	1	1,386	8	6:00/6:00	7:30/30:00
9	m	22	1	215	8	6:00/6:00	7:30/28:00
10	m	28	1	58	7	6:00/6:00	7:19/28:00
11	m	45	1	838	7	6:00/6:00	7:19/26:00
12	f	43	1	336	6	6:00/6:00	7:08/23:00

For the DCE-MRI examination, a single dose (0.1 mmol/kg of body weight) of gadopentetate dimeglumine (Gd-DTPA) was administered. Prior to Gd administration, Gd-dependent sequences, such as 3D T1-MPRAGE, were acquired. For the accurate conversion of signal intensity (SI) to CA concentration, a fast 3D VIBE sequence implemented six times, each time utilizing sequences using a different flip angle (FA) (5°, 10°, 15°, 20°, 25°, and 30°), was acquired multiple flip angles (mFAs). These six image sets of different FAs served as the base images for a post-processing calculation of a 3D T1 parametric image map (T10 map). Consequently, a conventional T1-w DCE-MRI perfusion protocol was implemented by utilizing a fast 3D VIBE sequence, with a repetition time (TR) of 7 ms and an echo time (TE) of 3.23 ms, while six baseline images were acquired before the injection of CA. The FA for the perfusion protocol was 15° (T1-w contrast). TR and TE parameters were selected in order to maximize the contrast-to-noise ratio (CNR) between normal and malignant tissues on *T*_1_-weighted images in brain. Analysis of DCE sequence was 512 × 512, 24 slices of 4 mm slice thickness. During the DCE protocol there were acquired 30 dynamic acquisitions with 11.3 s temporal resolution.

After the conventional DCE perfusion protocol (hereafter mentioned as the NoSnaps protocol), T2-wTSE, FLAIR, GRE, DWI, and 3D T1-MPRAGE sequences were obtained, as part of the routine MRI protocol for MS. In between these post-perfusion sequences, single T1-w 3D VIBE (FA = 15°) delayed acquisitions were acquired, using the same parameters as the perfusion protocol and lasting 11.3 s each. These delayed 3D T1-w VIBE sequences were incorporated in the conventional DCE protocol, while the intermediate intervals were computed by splines interpolation, and the resulting image set defined the extended DCE protocol (hereinafter called Snaps). Delayed DCE acquisitions, mFAs and conventional DCE perfusion were all acquired with the aforementioned fast 3D VIBE sequence, using TR 7 ms and TE 3.23 ms, 512 × 512 matrix size, 4 mm slice thickness and 24 slices.

**Figure 1:**
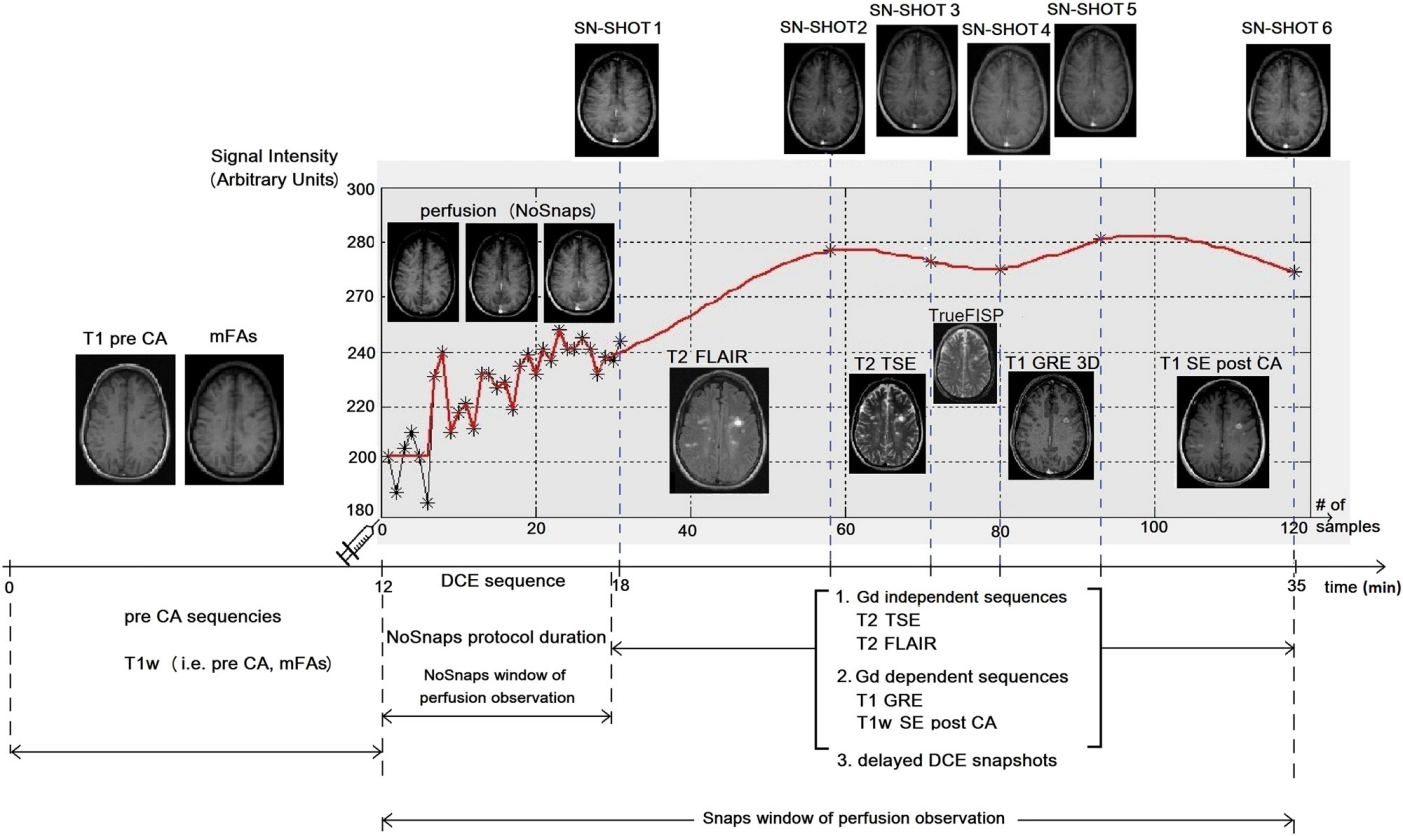
The time workflow of the MR protocol for patient number 2. Initially Gd-dependent sequences that do not need CA are acquired, i.e., mFA sequence. The DCE study starts at the 12th minute and lasts about 6 min, for the so-called NoSnaps protocol. Afterward, there were acquired Gd dependent (i.e., T1-w SE post CA) and independent (i.e., T2 FLAIR and DWI) sequences, while in between them there were acquired six delayed DCE snapshots. In the right part of the figure, an exemplary SI time curve is shown in which the first 30 asterisks are from the NoSnaps protocol, while the next six asterisks are the delayed DCE snapshots.

In Figure 1 an exemplary MRI protocol workflow for a patient is presented, including MR sequences prior to and after CA administration. An exemplary SI time curve of an enhanced pixel is shown in the right part of the figure. The asterisks in the time curve correspond to the time samples, the first 30 asterisks represent the NoSnaps acquisitions while the last six asterisks stem from the delayed T1-w acquisitions. By interpolating the delayed acquisitions with a splines algorithm in order to comply with the DCE sampling time (11.3 s), the complete Snaps protocol is obtained, depicted by the red fitted curve for an exemplary pixel inside the lesion regions of interest (ROIs).

The mean acquisition time for the Snaps protocol is extracted by calculating the mean number of Snaps acquisitions, multiplied by their duration of 11.3 s, and the result is added to the NoSnaps acquisition time, i.e., 6 min ([mean # of Snaps] × 11.3 s + 6 min). Overall, the acquisition time and the window time of perfusion observation for the NoSnaps protocol are 6 min for all patients, whereas the mean acquisition time for the Snaps protocol is 7 min and 21 s and the mean window time of perfusion observation after the interpolation is almost 26 min on average (Table 1). It is worth noticing the difference in time duration between the NoSnaps and Snaps protocols in the signal plot of Figure 1, where the window time of perfusion observation for the former protocol is 6 min and for the latter protocol is 23 min, by extending the MRI examination time in the Snaps protocol only by 1 min approximately.

An initial 3D volume of the DCE with a high CNR was used as a reference for the co-registration of the DCE dynamic series and the delayed DCE snapshots using FSL software [25,26], FLIRT, using the correlation ratio cost function and six degrees of freedom. ROIs were annotated by a neuroradiologist (EP) with 20 years of experience directly on the DCE sequence by consulting anatomical images, such as T1 post Gd and T2 FLAIR, including the part of the MS lesion with visible enhancement. Furthermore, a second set of ROIs was drawn including tissue outside the periphery of the enhancing part of the MS lesion, in order to compare the findings of the PK results in the active lesion area with the surrounding tissue.

### 2.2 PK models

All the examined lesions were analyzed using three different PK models: the well-established Tofts model (TM) [27], ETM [28], which considers the vascular contribution, and the Patlak model [29] that according to previous findings gives accurate results on small BBB leakage. For all these models, mFAs data, single T1-w acquisitions with different FAs, were used in order to accurately convert SIs into CA concentration. A population-averaged arterial input function from Weinmann et al. was used for analyzing the examinations [30]. Finally, estimated biomarkers were limited in physiological interpretable values *k* ^trans^ < 5 min^−1^, ve < 1, vp < 1.

### 2.3 Statistical analysis

*R*-squared (*R* ^2^) is a commonly used goodness-of-fit metric for a model. However, studies have shown that metrics that rely on the measurement of the absolute distance between the fitted curve and the given signal points are insufficient metrics in these problems [31]. To this end, *R* ^2^ can be biased when comparing models with different number of estimated parameters and given samples, as it inclines to favor the most complex ones. In this study, in order to consider the different number of time points for the NoSnaps and Snaps protocols, and the different number of the estimated biomarkers, from the three different PK models, the bias-corrected adjusted *R* ^2^ (adj-*R* ^2^) [32] was used instead of *R* ^2^. The model was penalized for extra parameters that do not contribute to explaining the variance, by the following equation:

(1) \text{adj-}{R}^{2}=1-\left(1-{R}^{2}\right)\left(n-1\right)/(n-k-1)

where *n* is the sample size and *k* is the number of predictors.

A statistical analysis was conducted in order to assess the fitting quality for every PK model – protocol combination (i.e., ETM–Snaps, TM–NoSnaps, etc.) using an iterative process described as follows: (a) all PK models were first grouped into groups A and B according to the protocol that was used for data acquisition (A and B were assigned to the data acquired using the Snaps and NoSnaps protocols, respectively), (b) an adj-*R* ^2^ threshold was applied varying from 0.1 to 0.5 regardless of the model, for every group in order to quantify regions with a subtle uptake from any of the three examined PK models

(2) \left(\text{adj-}{R}^{2}\_\text{ETM-protocol}\right)\text{OR}\left(\text{adj-}{R}^{2}\_\text{TM-protocol}\right)\text{OR}\left(\text{adj-}{R}^{2}\_\text{PATLAK-protocol}\right)\gt \text{threshold}

(c) pixels with adj-*R* ^2^ below the applied threshold for all the three examined PK models and belonging to the same protocol were excluded from the analysis, (d) afterward, a histogram analysis using the derived PK biomarkers was conducted, which resulted in several metrics such as mean, median, and several percentiles for every biomarker, and (e) the goodness of fit was examined from the resulting adj-*R* ^2^ by comparing both PK models and protocols.

Furthermore, the distribution of the resulting PK parameters after every thresholding was found to follow a non-normal distribution (*p*-value < 5%). A Wilcoxon–Mann–Whitney test was used in order to find significant differences among the two different perfusion protocols for every model. For this purpose, pairwise tests were applied to all parameters that have been calculated from all models but using a different perfusion protocol (i.e., *k* ^trans^_TM_Snaps and *k* ^trans^_TM_NoSnaps), which showed that there are no statistical dependencies (*p*-value < 5%), thus representing that the acquisition protocol affects the values of the fitted parameters.

The aforementioned statistical analysis was also applied to subsets of the original data, in order to examine the specific properties of each protocol separately and investigate their physiological interpretation. To this end, the range of biomarkers extracted from pixels identified by each of the two protocols (NoSnaps and Snaps) were examined and statistical measures were computed.

## 3 Results

**Figure 2:**
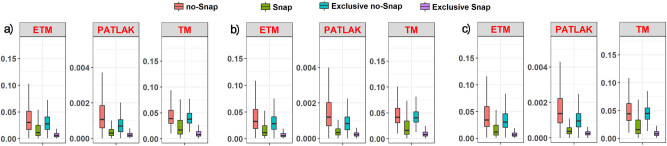
*k* ^trans^ (min^−1^) boxplots for different values of adj-*R* ^2^ thresholding: (a) 0.2 threshold, (b) 0.3 threshold, and (c) 0.4 threshold for the NoSnaps and Snaps protocols; pixels exclusively characterized by NoSnaps and pixels exclusively characterized by Snaps.

The range of *k* ^trans^ values for TM and ETM lies in the same range of values for the same protocol, while the Patlak model returns lower *k* ^trans^ values (Figure 1). Furthermore, when stricter thresholding was applied, a higher *k* ^trans^ range of values were achieved. This can be attributed to the fact that low enhancement pixels are represented by low fitting accuracy, due to the decreased SNR, so these are excluded in stricter thresholding. Moreover, a systematic decrease of the *k* ^trans^ values from the NoSnaps protocol to the Snaps protocol can be observed for every threshold, and the distribution of *k* ^trans^ boxplots on the Snaps protocol is skewed toward the higher *k* ^trans^ values compared with the NoSnaps protocol (Figure 2).

Subsequently, the number of pixels remaining after each consecutive thresholding that satisfied equation (2) was calculated for each protocol. At every threshold, the Snaps protocol resulted in a significant increase in the number of pixels that satisfy the thresholding condition as well as in the number of exclusively identified pixels (Table 2).

**Table 2:** Pixel contribution for every protocol after thresholding

Adj-*R* ^2^ threshold	Total number of pixels “NoSnaps”	Pixels identified exclusively from “NoSnaps”	Total number of pixels “Snaps”	Pixels identified exclusively from “Snaps”	Percentage difference on total pixels of Snaps compared to NoSnaps (%)
0.1	11,544	644	15,610	4,710	35
0.2	9,611	822	14,221	5,432	48
0.3	8,150	962	12,547	5,359	54
0.4	6,912	978	11,062	5,128	60

**Figure 3:**
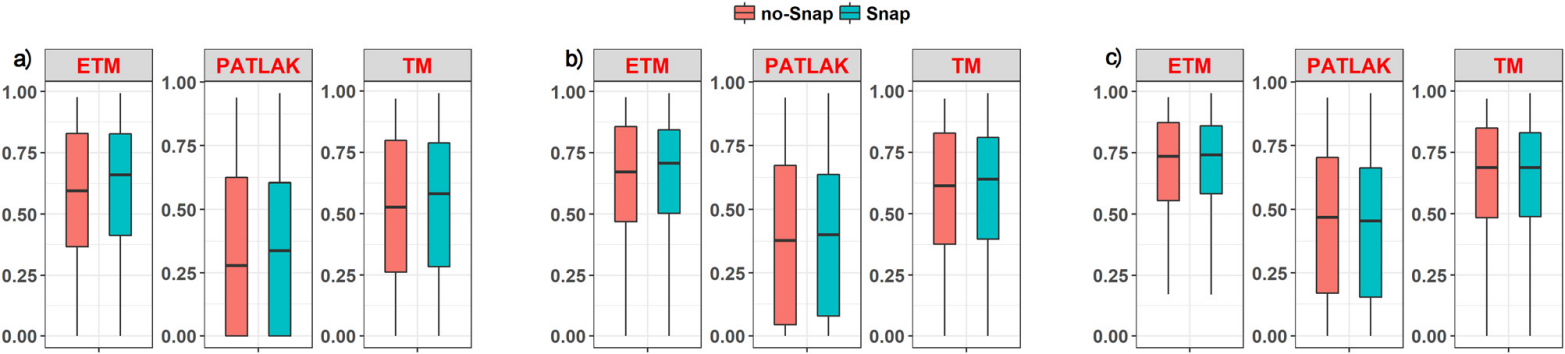
Adj-*R* ^2^ boxplots for different values of adj-*R* ^2^ thresholding: (a) 0.2 threshold, (b) 0.3 threshold, and (c) 0.4 threshold for NoSnap (red) and Snap (green) protocols.

Concerning the goodness of fit resulting from the two examined protocols, it can be noticed that the Snaps protocol led to increased fitting accuracies for all models and for each threshold (Figure 3). Moreover, it is apparent that the ETM model performs better in terms of fitting accuracy compared with the Tofts and Patlak PK models (Figure 3).

Moreover, it may be noticed from the SI time curve in the right part of Figure 1 that the signal from the conventional NoSnaps protocol starts from a baseline intensity of 200 and at the end of the protocol (30 time samples) the enhanced SI is almost 240, an increment of 20%. Examining the SI of the time-extended Snaps protocol, this also starts at the baseline value of 200 and after 120 samples ends at almost 280, which is an increment of 40%. It is obvious that for signals stemming from tissue with subtle BBB disruptions, thus low CA uptake, the time-extended SIs of the Snaps protocol result in a better SNR and thus a better fitting accuracy by a PK model. Finally, by observing the time curve during the extended perfusion time (Snaps), it may be noticed that there is a fluctuation in the signal. This can be attributed to the fact that after the NoSnaps protocol and in between the snapshots, a signal interpolation using a splines algorithm took place in a pixel-based approach.

Since the signal after the end of the conventional protocol was interpolated from 4 to 7 single measurements and because these measurements are contaminated by noise, the resulting time curve in the extended perfusion time follows the trend of these single measurements.

Regarding the second stage of statistical analysis, a similar procedure was followed in subsets of the original data, related to pixels identified exclusively by the NoSnaps and Snaps protocols, respectively, after each thresholding. Comparing the *k* ^trans^ boxplots of the overall pixels that each protocol quantified with the exclusively identified pixels of the same protocol, it is obvious that for every thresholding the excess of pixels that the Snaps protocol quantified are presented in the low *k* ^trans^ range of values. On the contrary, pixels that are identified only by the NoSnaps protocol are presented as dispersed in the range of values of the complete NoSnaps protocol (Figure 2).

**Figure 4:**
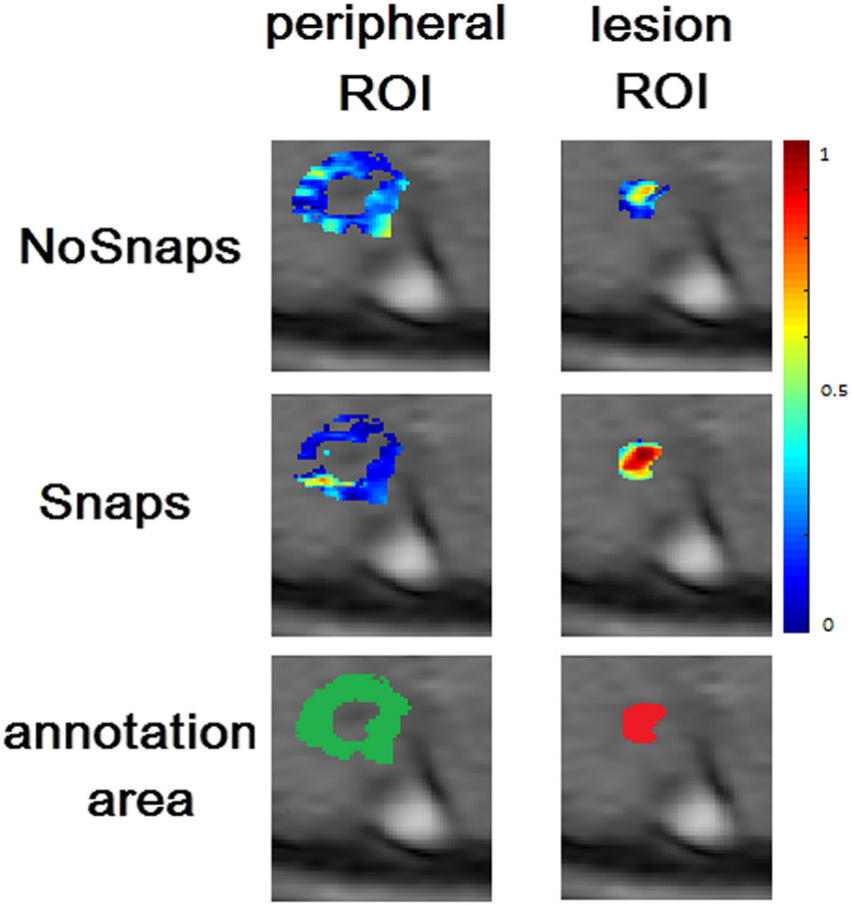
An exemplary figure depicting the adj-*R* ^2^ of the periphery of the lesion (first column) and the active lesion ROI (second column) for the NoSnaps (first row) and Snaps (second row) protocols using the ET model. In the third row, the annotation areas for the periphery of the lesion (green) and the active lesion (red) are depicted. Pixels that are not assigned with an adj-*R* ^2^ value and are included in the lesion or peripheral ROI are those pixels for which the fitting algorithm did provide reasonable perfusion parameters so they were excluded.

An additional analysis in order to validate the ROI areas was performed, by assessing the ETM goodness of fit (adj-*R* ^2^) in the lesion ROIs (foreground) as well as in ROIs annotated in the peripheral tissue (background). To this end, the initial lesion ROIs were dilated using an octagon kernel of size 15. Afterward, the lesion ROIs were removed from the dilated ROIs resulting in the peripheral ROIs, which were used in order to run a supplementary analysis, an exemplary result presented in Figure 4.

In more detail, the median values of the adj-*R* ^2^ were calculated for the active lesion and the peripheral tissue ROI, without considering any adj-*R* ^2^ threshold. Overall, the Snaps protocol not only achieved better goodness of fit in the ROIs of the active lesions compared to the NoSnaps (median [±s.d.] of NoSnaps adj-*R* ^2^ for every lesion is 0.324 [0.204], while for Snaps is 0.506 [0.192]) but also exhibited a larger absolute percentage change regarding the ROIs and corresponding peripheral tissue median adj-*R* ^2^ (abs[periphery-lesion]/lesion). Finally, for all the 24 examined lesions, the (absolute) median percentage change of goodness of fit from the lesion ROIs to the peripheral tissue was 61% for the Snaps protocol compared to 49% for the NoSnaps one.

## 4 Discussion

The BBB is a complex structure comprising endothelial cells with tight junctions, perivascular astrocytes, and pericyte vessels that separates the brain tissue from the circulating blood and prevents the entry of cells and toxic metabolites into the CNS [33]. Post-inflammatory BBB disruption, at the early stages of MS, might be caused by immune-active cells that penetrate the endothelial tight junctions and enter the cerebral tissue [8,9]. This transient breakdown of the BBB allows large hydrophilic substances, such as Gd-DTPA, to pass through the abnormal tight junctions or via pinocytosis [34] and accumulate locally in the affected brain parenchyma. These CAs have a shortening effect on the longitudinal relaxation time (*T*_1_), causing increased SIs in areas with BBB disruption. In the clinical setting, contrast-enhanced *T*_1_-weighted MRI is useful to detect focal BBB disruption in active MS lesions [35] and prove the dissemination of the disease in space and time, which is essential for making an early diagnosis of MS [36]. Although BBB leakage is more prominent in active focal lesions, increased BBB permeability has also been proved in diffuse NAWM areas [37,38], while there is also evidence of persistent BBB abnormalities in chronic inactive lesions [16]. Since the impairment of BBB function is of vital importance for the pathogenesis of MS, many treatment strategies target the resolution of inflammation and protection of BBB function [1,2]. Consequently, the detection and accurate quantification of the BBB permeability are very important for the diagnosis, determination of disease activity and estimation of treatment efficacy in MS.

To this end, the previously presented time-extended DCE-MRI protocol called Snaps, tailored for quantifying subtle BBB disruption, is proposed, providing extended temporal perfusion information, at the expense of minimally extending the conventional DCE acquisition time. Furthermore, the Snaps protocol combined with the extended Tofts PK model resulted in better spatial characterization of the MS-enhancing lesions, in terms of quantifying a significantly greater number of pixels with adequate fitting accuracy. Most importantly, these pixels were found to lie in the low *k* ^trans^ range, indicating improved detection and quantification of even subtle BBB disruption in patients with RRMS.

In previous studies that examined the dynamics of late enhancement on MS lesions [21,22,23], time-extended DCE protocols were utilized in patients with MS, with the acquisition time extending up to 155 min [23]. These time-consuming protocols could not easily be applied in clinical practice. On the contrary, the proposed time-extended DCE-MRI protocol extended the window of perfusion observation by 17 min on average, by minimally prolonging the examination time, 1 min on average. Other studies have been concentrated on finding subtle BBB disruption in NAWM [6,7,18,38,39,40] and GM [7] of patients with MS or on visibly non-enhancing MS lesions [6,18,19,20,22], pointing out the importance of quantifying these non-visible BBB disruptions by PK modeling. In the current study, a similar approach was followed by trying to quantify more accurately even non-visible parts of the enhancing MS lesions.

Other studies investigated the optimal protocol and acquisition parameters in combination with model selection for the accurate quantification of low BBB permeabilities. Both Cramer et al. [5] and Barnes et al. [14] found that the Patlak model is the most accurate for low BBB leakage quantification under certain circumstances. More specifically, the Patlak model is able to accurately measure low BBB disruptions when back diffusion is ignored, and thus the total measurement duration considering the permeability of the lesion that is measured plays a key role in the accuracy and precision of the results. Moreover, Barnes et al. [14] concluded that baseline acquisition should be long enough, 1–4 min, in order to achieve accurate permeability estimation on the Patlak model. Both studies also reported that when using Patlak to quantify low permeabilities (*k* ^trans^ < 2 × 10^−3^  min^−1^), increased acquisition times (>15 min) will significantly improve the measurement accuracy.

Sampling rate is another parameter that may influence the accuracy of the measurement, so one should take into account the lesion that will be quantified, the PK model that will be used and the fact that an enhanced lesion usually presents high variation in the very first samples, after the CA injection, and afterward there is a medium to low variation in the SI. Jelescu et al. [15] proposed a dual temporal resolution protocol that is described by high temporal resolution and low spatial resolution in the first minute of perfusion in order to efficiently capture the first-pass bolus. For the following 20 min, protocol resolution changes to low temporal and high spatial resolution in order to ensure accurate detection and segmentation of even small MS lesions. This protocol was also used by van de Haar et al. [41] to study subtle BBB leakages appearing in the neurodegenerative Alzheimer’s disorder, while in the same study it was reported that shorter scan times can lead to significantly overestimated permeabilities in lesions that are described by low BBB leakage.

Comparing the methodology presented herein to those of previous studies [21,22,23], a quantitative perfusion protocol is utilized that is able to quantify even subtle BBB leakage and is not based on a visual inspection of the SI changes that provide a binary result (i.e., enhancing or not) or *T*_1_ relaxation times that still are prone to inaccuracies biased from inherent protocol parameters. Moreover, compared with the previously reported dual temporal resolution protocol [15], the proposed method in this study satisfactorily captured the first-pass bolus considering a sufficient temporal resolution of 11.3 s without compromising the spatial resolution in the first minute of the perfusion and preserving high spatial resolution, 512 × 512 pixels, for the entire duration of the perfusion. The methodology presented herein resulted in permeabilities close to values that prior studies reported [5,25]. Additionally, the previously reported [41,42] overestimation of permeabilities as well as the skewness of boxplots to higher *k* ^trans^ values when using short scan times (Figure 2) are also confirmed. Moreover, permeabilities obtained from the Patlak model are significantly decreased compared to those from TM and ETM, in-line with previous findings [5,15]. Finally, previously reported model and acquisition parameters for measuring subtle BBB disruptions are achieved, considering that in this study the time of perfusion observation was kept long enough (average 26 min), the baseline acquisition was about 1 min, and the temporal resolution was 11.3 s, an adequate interval in order to efficiently capture the first-pass bolus dynamics.

Regarding the model selection part of this work, considering that active lesions are being studied and the fact that these lesions might have different degrees of BBB disruptions, it cannot be reported that only subtle BBB disruptions are being measured. In contrast, using this methodology and considering the time-extended perfusion protocol, it is reported that the low BBB permeabilities can also be accurately quantified, providing a more precise identification of the aforementioned lesions. Finally, considering that fitting accuracy is a metric of goodness of fit, as well as the fact that higher fitting accuracy indicates better reliability of the measurement, this method relied on selection criteria among different PK models and DCE protocols that were based on the amount of pixels that a model–protocol combination resulted after a thresholding procedure. Thus, under the assumption that higher fitting accuracies imply a better analysis method and considering the fact that this study was based on the adj-*R* ^2^, a metric that is independent of the different number of samples among the different protocols (Snap and NoSnap), and the different number of estimated parameters among the different PK models (TM, ETM, and Patlak), it can be deduced that the findings of the present study will not introduce a bias in the selected protocol and PK model analysis.

The principal limitation of the current work is the limited size of the patient population, which may be inadequate in order to draw a definite conclusion concerning the added-value of the proposed method. Future work in this direction needs to further investigate the accuracy of the current methodology using larger and more diverse patient cohorts. Moreover, in future work, visibly non-enhancing lesions and NAWM regions can be examined with the aforementioned methodology, in order to examine the subtle BBB disruptions in these areas. Considering the methodology of this work, the interpolation method for embedding the Snaps protocol into the conventional perfusion protocol could be replaced with a more robust method in order to exclude inaccuracies in the intermediate time samples. Finally, the methodology presented herein could be applied to other pathologies in order to extract information from additional perfusion biomarkers and investigate the reproducibility of the current method, in cases where late enhancement can reveal critical pathophysiological processes, such as in brain tumors.

## 5 Conclusion

DCE-MRI is a major imaging technique for BBB leakage quantification in MS lesions. Considering reports from previous studies, most MS lesions are described by low BBB permeabilities compared with tumors, necessitating the requirement of increased acquisition time on the perfusion studies. Longer scan time implies increased number of measurements (samples) and thus more time for the CA to extravasate to the measured tissue. This indicates higher tissue concentrations that is of major importance when trying to measure subtle BBB disruptions. Furthermore, considering the necessity of minimizing the patient examination time in the MR system, it is of great importance to provide new methods that extend the time of perfusion observation without charging the examination time. In this study we compared three different PK models and two DCE-MRI protocols, and by measuring even subtle BBB disruptions, we achieved a better spatial characterization and quantification of the enhancing MS lesions with a minimal extension of the MRI acquisition time.
